# Neonicotinoid-Induced Cytotoxicity: Insights into Cellular Mechanisms and Health Risks

**DOI:** 10.3390/toxics13070576

**Published:** 2025-07-09

**Authors:** Yuqing Ma, Qiangwei Wang

**Affiliations:** Ministry of Agriculture Key Laboratory of Molecular Biology of Crop Pathogens and Insects, Zhejiang Key Laboratory of Biology and Ecological Regulation of Crop Pathogens and Insects, Institute of Pesticide and Environmental Toxicology, Zhejiang University, Hangzhou 310058, China

**Keywords:** neonicotinoids, cytotoxicity, oxidative stress, mitochondrial dysfunction, human health risk

## Abstract

Neonicotinoids are extensively used in agricultural production, yet increasing evidence highlights their cytotoxic effects on various cell types. Research has demonstrated that these pesticides can significantly impair the viability and function of reproductive, adipose, neural, immune, and epithelial cells. The underlying mechanisms involve metabolic disturbances, mitochondrial dysfunction, and oxidative stress. These cellular effects raise serious concerns about the potential risks neonicotinoids pose to both human health and the environment. Further investigation is essential to fully understand their toxicological impact and to inform safer pesticide regulation and use.

## 1. Introduction

Neonicotinoid insecticides emerged in the 1990s as a revolutionary class of crop protection chemicals, with imidacloprid first introduced in 1991 by Bayer CropScience. Their development represented a significant breakthrough in addressing pest resistance issues while offering reduced environmental persistence compared to organochlorine compounds and lower acute mammalian toxicity than organophosphates [1]. By 2022, neonicotinoids accounted for approximately 25% of the global insecticide market, with annual sales exceeding $1.5 billion [2].

**Figure 1:**
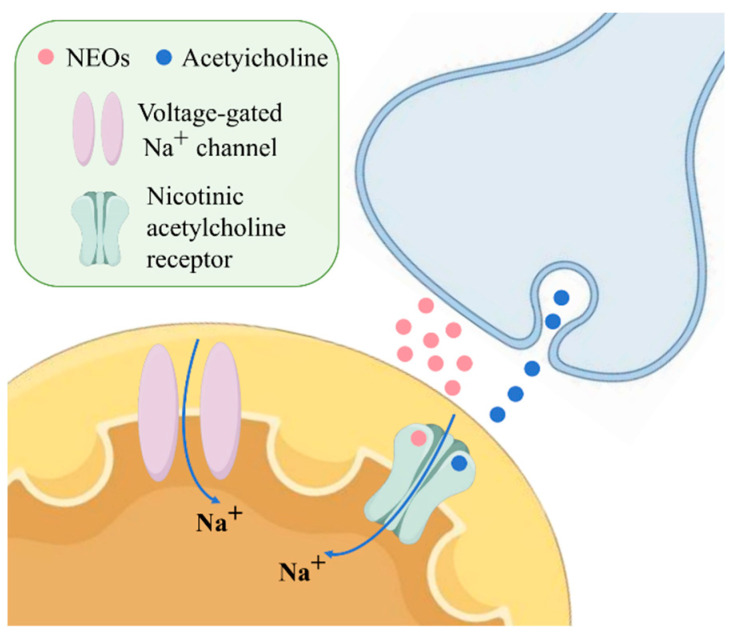
Action mechanism of NEOs.

The major commercially available neonicotinoids include imidacloprid (IMI), thiamethoxam (TMX), thiacloprid (THI), nitenpyram (NIT), acetamiprid (ACE), clothianidin (CLO), dinotefuran (DIN), and the related compounds flupyradifurone (FLU) and fipronil (FIP), with varying usage patterns across different regions [3]. The main regions of neonicotinoid use are Latin America, Asia, and North America (75% of total use). Neonicotinoids are broadly approved and widely used in China (with some ecological use restrictions), subject to conditional registrations and partial use limitations in the United States, and largely banned in the European Union—with the exception of ACE and a few newer analogs [4]. These compounds act primarily through agonistic binding to nicotinic acetylcholine receptors (nAChRs), causing continuous stimulation and disruption of neural signaling that ultimately leads to paralysis and death in target insects [5]. The specific mechanism of action is illustrated in Figure 1. The selective toxicity of neonicotinoids toward insects over mammals stems from their higher binding affinity for insect nAChRs, attributed to differences in receptor subunit composition and binding site properties [6].

Despite their initial portrayal as environmentally friendly alternatives, neonicotinoids have increasingly been recognized as emerging contaminants of concern due to their widespread environmental distribution, persistence in soils and water systems, and demonstrated toxicity to non-target organisms [7]. Regulatory responses have varied globally, with the European Union implementing significant restrictions on several neonicotinoids in 2013 due to pollinator health concerns, while their use continues extensively in many other regions, including the United States, China, and Brazil [8]. Ecotoxicological studies have established a clear hierarchy of species sensitivity to neonicotinoids, with insects showing the highest susceptibility (LC_50_ 0.01–2.38 mg/L; honey bees as low as 3.7–81 ng/L), followed by crustaceans (LC_50_ 0.59–37.75 mg/L), fish (LC_50_ 1.2–241 mg/L), birds (LD_50_ 15–>2000 mg/kg), and mammals (LD_50_ 82–>5000 mg/kg) [9]. This spectrum of toxicity initially supported the perception of low mammalian risk. However, a growing body of evidence challenges this assumption, documenting mammalian toxic effects including neurodevelopmental disruption, immunotoxicity, hepatotoxicity, and nephrotoxicity following acute and chronic exposures [10,11,12,13,14]. Epidemiological studies have further suggested potential links between neonicotinoid exposure and adverse human health outcomes, raising substantial public health concerns [15,16].

In vitro approaches have gained prominence for assessing chemical hazards, offering ethical, economical, and mechanistically informative alternatives to traditional animal testing. Cellular models provide valuable insights into toxicity mechanisms with high throughput capabilities and reduced costs compared to in vivo studies. Cytotoxicity assessments can reveal diverse biological responses, including cell viability changes, metabolic disruptions, oxidative stress, genotoxicity, and alterations to critical cellular pathways. These methodologies have been increasingly applied to neonicotinoid research, elucidating subcellular mechanisms of toxicity that may not be readily apparent in whole-organism studies [17,18]. Despite numerous studies examining individual aspects of neonicotinoid cytotoxicity, a comprehensive mechanistic understanding remains elusive, with significant knowledge gaps regarding cell-type specific responses, exposure thresholds, mixture effects, and the relevance of in vitro findings to human health risk assessment. This review synthesizes current research on neonicotinoid-induced cytotoxicity, focusing on molecular mechanisms of action, cell-type specific effects, dose-response relationships, and implications for environmental and human health. We critically examine methodological approaches, identify research gaps, and discuss how emerging cellular insights can inform risk assessment and regulatory frameworks for this important class of pesticides. To provide context for the subsequent mechanistic and toxicological discussions, Table 1 summarizes the main neonicotinoid compounds, their typical formulations.

## 2. The Molecular Mechanism of Neonicotinoids Inducing Cytotoxicity

### 2.1. Oxidative Stress Induced by Neonicotinoids

Neonicotinoid compounds undergo metabolic conversion within cells, leading to excessive production of reactive oxygen species (ROS) as byproducts. Neonicotinoids and their formulations can induce an overproduction of ROS, including superoxide anions, hydrogen peroxide, and hydroxyl radicals [19]. For example, imidacloprid exposure (100 μM, 24 h) has been shown to increase ROS levels 2.5-fold in HepG2 cells while simultaneously reducing superoxide dismutase (SOD) activity by 30% [3]. THI (480SC, 120 μg/mL, 4 h) causes oxidative stress damage in bovine lymphocytes, with ROS level increasing 2-fold, and protein carbonylation level also increasing [20]. Neonicotinoids induce oxidative stress through multiple interconnected mechanisms, thereby disrupting cellular redox homeostasis. For instance: cytochrome P450 enzymes (especially CYP3A4 and CYP2C19) metabolize neonicotinoids into reactive intermediates, such as nitroso-guanidine compounds that directly generate ROS [21]. TMX (≥50 μM) significantly inhibits mitochondrial complexes I and III, disrupting electron transport chain function and increasing superoxide anion production, with exposed SH-SY5Y cells showing 40% reduction in mitochondrial membrane potential and 25% decrease in ATP production [22]. As a neuron-like cell line, SH-SY5Y cells may be more susceptible to mitochondrial dysfunction than hepatocytes, thereby influencing downstream ROS production. CLO severely compromises antioxidant defense systems, with 10 mg/kg doses reducing SOD activity by 45%, catalase activity by 38%, and glutathione peroxidase activity by 52% in mouse hepatocytes after 7-day treatment [23,24]. This multifaceted assault on cellular redox systems ultimately leads to oxidative damage to lipids, proteins, and nucleic acids, initiating downstream cytotoxic responses. Although oxidative imbalance is consistently observed, few studies have directly compared commercial formulations with pure active ingredients, which may underestimate the synergistic or additive toxicity contributed by formulation adjuvants.

### 2.2. DNA Damage and Genotoxic Effects of Neonicotinoids

Neonicotinoid pesticides induce DNA damage and may compromise genomic stability through multiple interconnected mechanisms. One major pathway involves oxidative stress, as evidenced by a 3-fold increase in 8-hydroxy-2′-deoxyguanosine (8-OHdG) levels and a 4.2-fold rise in comet assay tail moment following IMI (20 μM, 48 h) exposure in human lymphocytes, indicating substantial oxidative DNA damage [25]. This oxidative stress is driven by ROS generated during neonicotinoid metabolism, which directly attack DNA bases and the sugar-phosphate backbone, leading to strand breaks and mutations. In addition to direct oxidative damage, neonicotinoids impair the DNA repair process, further exacerbating genomic instability. IMI (35% suspension concentrate, 35SC) exposure caused DNA damage in zebrafish gill cells, which show a highly significant difference in the genotoxic effect compared to zebrafish not exposed to pesticides [26]. Furthermore, exposure to high-dose IMI (200 μM, 24 h) increases micronuclei frequency by 2.8-fold in CHO cells, demonstrating its genotoxic potential through chromosomal abnormalities [27]. Previous studies demonstrated that CLO induces significant oxidative stress and causes both single- and double-strand DNA breaks in human bronchial epithelial cells (BEAS-2B), with persistent DNA damage even after recovery periods [28]. These findings suggest a potential compromise in DNA repair capacity, although the specific involvement of ATM/ATR remains to be elucidated. These findings highlight the multifaceted impact of neonicotinoids on genomic integrity by inducing oxidative DNA damage, disrupting repair mechanisms, and promoting chromosomal instability.

### 2.3. Neonicotinoid-Induced Cell Apoptosis: Pathways and Evidence

Neonicotinoids induce programmed cell death through both receptor-mediated mechanisms and intrinsic apoptotic pathways, disrupting cellular homeostasis and triggering caspase activation. In SH-SY5Y neuronal cells, CLO (10 μM, 48 h) exposure leads to a 56% reduction in α7-nAChR expression, disrupting calcium signaling and activating caspase-3, a key executioner of apoptosis [29]. Similarly, IMI (50 μM, 24 h) exposure in hepatocytes elevates the Bax/Bcl-2 ratio 3.5-fold, promotes cytochrome c release by 2.8-fold, and enhances caspase-9 activity 4-fold, indicating activation of the intrinsic mitochondrial apoptotic pathway [30]. Neonicotinoids can also engage the extrinsic apoptotic pathway through death receptor signaling. CLO (150 μM, 24 h) exposure significantly upregulates Fas receptor expression (2.2-fold) and increases caspase-8 activity (3.6-fold), highlighting its role in receptor-mediated apoptosis [31]. Moreover, neonicotinoid-induced apoptosis is dose-dependent, with low concentrations (<10 μM) exhibiting minimal effects, while higher doses (50–100 μM, 72 h) lead to substantial neuronal cell death, with 30–45% apoptotic rates observed [32]. Moreover, CLO treatment led to a decrease in mitochondrial membrane potential, release of cytochrome c, elevated ROS levels, and activation of caspase-3 and -9 in Caco-2 cells, suggesting that it induces apoptosis via the mitochondrial pathway [33]. Methodological differences in the assessment of apoptosis, such as variations in caspase activity measurement, mitochondrial membrane potential analysis, and receptor expression profiling, may affect the comparability of results across studies. Notably, these apoptotic effects exhibit cell-type specificity, with neuronal cells displaying greater sensitivity to neonicotinoid exposure at comparable doses. This heightened vulnerability may be attributed to differences in receptor expression or mitochondrial resilience in neuronal cells. Collectively, these findings indicate that neonicotinoids disrupt apoptotic processes through both mitochondrial and receptor-mediated pathways, leading to cell-type-dependent cytotoxicity.

### 2.4. Endoplasmic Reticulum Stress Response Triggered by Neonicotinoid Exposure

Neonicotinoids induce endoplasmic reticulum (ER) stress, playing a crucial role in their cytotoxic effects. Exposure to IMI (≥75 μM, 12 h) in mouse hepatocytes leads to a 2.4-fold increase in glucose-regulated protein 78 (GRP78) expression and a 3.1-fold elevation in CCAAT-enhancer-binding protein homologous protein (CHOP) levels, indicating a pronounced ER stress response [34]. A study found that acetamiprid induces ER stress in SH-SY5Y neuroblastoma cells, as evidenced by increased protein levels of IRE1α and GRP90, along with upregulated gene expression of caspase-3, -4, and -9, suggesting activation of the unfolded protein response (UPR) pathway and subsequent induction of apoptosis [35]. Furthermore, activation of the IRE1α-XBP1 pathway was observed to be associated with mitochondrial dysfunction, providing additional evidence for the critical role of UPR in cellular cytotoxicity [36]. In a rat model, 30-day exposure to IMI (45 mg/kg/day) caused pancreatic damage, with elevated ER stress markers (IRE1α, XBP1, CHOP), oxidative stress, and inflammation. Lycopene co-treatment alleviated these effects, suggesting a protective role [37]. In mice, TMX exposure reduced ovarian weight, disrupted hormone receptor expression, and impaired follicle development. Transcriptomic and molecular analyses confirmed increased GRP78 and CHOP levels, linking oocyte quality decline to ER stress [38]. In Mauremys reevesii, thiacloprid exposure triggered oxidative and ER stress in the liver, inducing apoptosis. Elevated stress markers indicated liver dysfunction via ER stress pathways [39]. Current studies on this process exhibit considerable variability in terms of model organisms, tissue types, and biomarkers assessed. Moreover, most investigations have focused primarily on a limited set of ER stress markers, such as GRP78 and CHOP, without systematically evaluating the three canonical branches of the unfolded protein response (UPR), including PERK, ATF6, and IRE1α, thereby limiting mechanistic insight. In addition, the temporal sequence of ER stress in relation to other toxicological events, such as oxidative stress and apoptosis, remains unclear, and causal relationships have yet to be elucidated. Nonetheless, existing evidence has preliminarily demonstrated that neonicotinoids can activate UPR pathways, leading to mitochondrial dysfunction, inflammatory responses, and cell death.

### 2.5. Disruption of Calcium Homeostasis in Cells by Neonicotinoids

Studies have demonstrated that CLO induces a transient elevation of intracellular calcium concentration in human neuroblastoma SH-SY5Y cells and activates the ERK1/2 signaling pathway, suggesting that neonicotinoids may affect neuronal cellular functions via nAChR-mediated calcium signaling [40]. In vivo studies using mice have shown that neonicotinoid exposure can activate store-operated calcium entry (SOCE) in hepatic cells, leading to calcium overload, mitochondrial bioenergetic dysfunction, and cell cycle arrest at the S phase [12]. In aquatic species, similar disturbances in calcium balance have been observed. For instance, goldfish exposed to TMX and dinotefuran exhibited a marked decrease in plasma calcium concentration during the early stages of exposure, accompanied by increased scale loss, highlighting systemic physiological stress [41]. At the neural level, neonicotinoids have been shown to interfere with calcium regulatory mechanisms by activating voltage-gated calcium channels (VGCCs), inhibiting calcium pumps such as PMCA, and disrupting endoplasmic reticulum calcium stores. These alterations result in sustained intracellular calcium elevations, which can trigger oxidative stress, neuroinflammation, and ultimately, neuronal cell death [42]. Moreover, excessive calcium influx induced by compounds such as IMI and TMX can activate calcium/calmodulin-dependent kinases in cerebellar neurons, promoting hyperphosphorylation of tau protein. This mechanism is closely associated with the pathogenesis of neurodegenerative diseases, including Alzheimer’s disease [43]. Current studies on neonicotinoid-induced disruption of calcium homeostasis have revealed that these compounds affect both neuronal and non-neuronal systems through multiple mechanisms, including activation of voltage-gated calcium channels, inhibition of calcium pump activity, and disruption of endoplasmic reticulum calcium stores. However, the specific contributions of these mechanisms exhibit substantial heterogeneity across species, tissue types, and experimental designs, limiting the ability to formulate a unified mechanistic framework. Notably, several studies have linked calcium dysregulation to key molecular events associated with neurodegenerative diseases, such as tau protein hyperphosphorylation, suggesting a potential neurotoxic risk of neonicotinoid exposure.

### 2.6. Immunotoxicity Mechanisms Mediated by Neonicotinoids

Neonicotinoids exert immunotoxic effects by disrupting immune cell function and inflammatory signaling pathways, ultimately compromising host defense mechanisms. Studies have shown that the neonicotinoid insecticide IMI significantly suppresses the phagocytic activity of human macrophages and *Drosophila* hemocytes, with a more pronounced effect on *Drosophila* cells, particularly under non-immune-activated conditions, indicating a higher sensitivity and immunotoxic potential in invertebrate immune systems [44]. IMI (25 μM, 48 h) significantly impairs innate immune responses by reducing macrophage phagocytic capacity by 38% and decreasing nitric oxide production by 45%, weakening the ability to combat pathogens [45]. This immunosuppression is closely linked to pro-inflammatory cytokine dysregulation, as exposure to TMX (60 μM, 24 h) leads to a marked increase in TNF-α, IL-1β, and IL-6 levels (2.7-fold, 3.2-fold, and 2.4-fold, respectively) through NF-κB pathway activation in macrophages [46]. In addition to cytokine imbalances, neonicotinoids also suppress adaptive immune responses, as shown by CLO (30 μM, 72 h) exposure, which inhibits T-cell proliferation by 55% and reduces IL-2 secretion by 62% following mitogen stimulation, thereby impairing immune activation [23]. These effects highlight the immunosuppressive potential of neonicotinoids, increasing host vulnerability to infections and inflammatory disorders. Current studies indicate that neonicotinoid pesticides exert significant immunosuppressive effects by disrupting both innate immune cell phagocytic function and pro-inflammatory cytokine regulation, as well as inhibiting adaptive immune responses, such as T cell proliferation and cytokine secretion, ultimately compromising host defense mechanisms. However, the relative sensitivities and interactions between innate and adaptive immune systems remain unclear, and heterogeneity in experimental models and assay methodologies further complicates the interpretation of these findings.

## 3. Cell Type Specific Response and Organ System Toxicity of Neonicotinoids

### 3.1. Neurotoxicity of Neonicotinoids in Neuronal Cells

Neuronal cells exhibit heightened sensitivity to neonicotinoid exposure due to their expression of nAChRs, with CLO demonstrating an 8-fold higher affinity for neuronal nAChRs compared to peripheral ones, leading to sustained receptor desensitization and calcium dysregulation [18]. Numerous studies have investigated the neurotoxic effects of neonicotinoids across various neuronal cell models. For instance, an in vitro study demonstrated that commercially available insecticides IMI (20% concentrated suspension, 20CS) and ACE (20% soluble powder, 20SP) significantly increased the production of ROS and reactive nitrogen species (RNS) in SH-SY5Y cells, leading to mitochondrial dysfunction and early apoptosis, suggesting potential neurotoxic risks of these insecticides to human neuronal cells [47]. According to Öztaş et al., ACE exhibits a clear dose-dependent cytotoxicity in SH-SY5Y neuroblastoma cells. High concentrations of ACE strongly inhibit cell viability [35]. Moreover, ACE significantly induces ROS generation in a dose-dependent manner. These findings indicate that the neurotoxicity of ACE is not only reflected by the reduction in cell viability, but is also closely associated with oxidative stress responses. Cheng et al. studied the combined toxicity of IMI, ACE, and TMX using a human neuroblastoma cell line (SK-N-SH) [48]. They found that binary and ternary mixtures could enhance the inhibition of the growth of SK-N-SH cells at low doses; specifically, all the mixtures showed synergistic effects at concentrations < 50 mg/L. Choosing lund human mesencephalic (LUHMES) as the target, Loser et al. investigated a subset of six neonicotinoids, namely ACE, IMI, CLO, THI, TMX and DIN, and they determined pEC_25_ values [49]. The results demonstrated that these neonicotinoids induced alterations in intracellular calcium signaling in neurons, indicating their disruptive effects on neuronal function. Furthermore, these effects were blocked by specific antagonists, further confirming their neurotoxic potential. Wang et al. found that exposure to IMI and ACE caused metabolic disturbances and redox homeostasis damage in Neuro-2a cells, with ACE at 267.1 μM exhibiting the most significant cell membrane damage and markedly inducing caspase 7 expression, suggesting that ACE exerts toxicity through promoting necrosis and apoptosis [50]. Annabi et al. investigated the toxic effects of ACE in PC12 cells and reported a significant decrease in cell viability assessed by the MTT assay [51]. They also observed that ACE induced ROS generation, followed by lipid peroxidation, as indicated by increased malondialdehyde (MDA) levels. The increase in cell death was accompanied by a reduction in mitochondrial membrane potential. Eriksson et al. studied the cytotoxicity of IMI (70% water dispersible granule, 70WG) on human glial cells by treating D384 astrocytes with various concentrations of IMI, revealing a significant reduction in DNA content in cells exposed to 0.8 mM IMI, indicating a decrease in cell numbers following treatment [52]. These neurotoxic effects manifest as cognitive deficits in the central nervous system, as seen in mice exposed to IMI (10 mg/kg/day, 28 days), which exhibited a 35% longer latency in spatial memory tasks and a 42% reduction in hippocampal neurogenesis. A 2021 systematic review evaluated the biochemical and behavioral effects of neonicotinoids on the mammalian nervous system. The study found that early-life exposure to neonicotinoids disrupts normal neural development, leading to reduced neurogenesis, abnormal neuronal migration, and the induction of neuroinflammation. In adulthood, neonicotinoids induce neurobehavioral toxicity through modulation of nAChRs, characterized by decreased nAChR expression, altered acetylcholinesterase activity, and dysfunction of the nigrostriatal dopaminergic system, ultimately resulting in oxidative stress, neuroinflammation, and neuronal cell death [5]. Although existing studies have revealed the neurotoxicity and potential mechanisms of neonicotinoid pesticides on neuronal cells, they have significant limitations. Most research is based on single cell lines and acute high-dose exposures, and studies on combined toxicity remain insufficient, overlooking the complexity of multi-pesticide co-exposure in real environmental settings. Future research should adopt long-term, multi-dose exposure designs that better reflect human exposure levels, incorporate multiple cell types and 3D neural models, and integrate pharmacokinetic and behavioral studies to provide a more comprehensive assessment of the neurotoxic risks posed by neonicotinoids.

### 3.2. Reproductive Toxicity of Neonicotinoids in Reproductive Cells and Tissues

Distinct responses to neonicotinoid exposure were observed in gametes and reproductive tissues. In toxicity research focusing on reproductive cells, Ibrahim et al. found that IMI at 400 μM significantly affects the rat leydig cell line (LC-540), leading to decreased cell viability, disorganization of cytoskeletal filaments, and perinuclear aggregation, along with the presence of cytoplasmic vacuoles, autophagic vacuoles, lysosomal damage, and mitochondrial dysfunction [53]. TMX exposure induces meiotic arrest, chromosome misalignment, and spindle abnormalities in bovine oocytes, thereby compromising oocyte quality. Additionally, it triggers oxidative stress, mitochondrial dysfunction, and apoptosis, ultimately reducing the developmental potential of embryos [54]. In the female reproductive system, ACE evokes pathomorphological alterations in follicles. In vivo studies showed that female rats exposed to IMI (10 mg/kg/day, 60 days) experienced a 35% reduction in ovarian follicle count, a 28% decrease in corpus luteum number, and a 42% lower serum estradiol level, while exposed males exhibited a 31% decrease in sperm count, a 44% reduction in testosterone, and significant histopathological changes in seminiferous tubules [55,56]. 2-cell stage mice embryos were cultured in media with various concentrations of active compounds THI, TMX, ACE and CLO until blastocystall, and results showed that, neonicotinoids at concentration of 100 μM negatively affected embryonic development. THI impairs development and quality of both mouse and rabbit preimplantation embryos. THI and ACE also decreased quality of blastocysts at 10 μM concentration [57]. Other studies have further explored the mechanistic basis of male reproductive toxicity, revealing that IMI exposure impairs testicular and sperm function in male rats, as evidenced by oxidative imbalance, increased DNA fragmentation, reduced sperm quality, and enhanced apoptosis, indicating significant developmental reproductive toxicity [58]. Additionally, TMX and ACE exposure in mice reduced ovarian weight, along with metabolic changes, disrupted hormone receptor expression, increased granulosa cell apoptosis, and impaired oocyte quality by inducing oxidative and endoplasmic reticulum stress [38,56]. TMX (75% water dispersible granule, 75WG) exposure impairs male reproductive function by reducing testosterone, damaging sperm parameters, and inducing oxidative DNA damage in testicular tissue [59]. These findings demonstrate the significant effects of neonicotinoids on reproductive health and point to the need for more research to clarify the underlying mechanisms of their developmental reproductive toxicity.

### 3.3. Hepatotoxic and Nephrotoxic Effects of Neonicotinoids on Hepatocytes and Renal Tubular Cells

Neonicotinoid exposure has been shown to cause significant toxic effects in both hepatocytes and renal tubular cells, which play crucial roles in the metabolism and excretion of these compounds. IMI treatment resulted in cytotoxicity in the HepG2, starting at concentrations of 0.5 mM (24 h) and 0.25 mM (48 h), and reducing cell viability from 0.5 mM onwards (24 and 48 h). IMI significantly decreased the mitochondrial membrane potential at both time points investigated (2.0 mM), and also induced damage to the cell membrane [60]. However, as HepG2 is a transformed hepatic carcinoma cell line, its metabolic and redox characteristics may differ markedly from normal hepatocytes, potentially influencing susceptibility and limiting extrapolation. Li et al. found that IMI exposure induced autophagy accompanied by advanced autophagy markers BNIP3, Beclin1, and LC3II/I in CIK cells, reduced the levels of miR-451, increased the expression of Cab39 and AMPK, inhibited AKT/mTOR signaling, and activated the JNK pathway [61]. Several studies have also investigated the effects of IMI (17.8WG) on the liver of rats. One study reported that imidacloprid treatment caused dilation of the central vein and hepatic venules, as well as expansion of the sinusoidal spaces between hepatocytes [62]. Another study demonstrated that IMI exposure increased oxidative stress in the rat liver, characterized by decreased activities of superoxide dismutase (SOD) and glutathione peroxidase (GPx), along with reduced glutathione (GSH) levels [63]. Furthermore, a 90-day oral toxicity study on female rats revealed that imidacloprid administration led to central vein dilation, hepatocellular degeneration, and an increase in liver weight, indicating significant physiological, biochemical, and histopathological alterations [64]. In addition to IMI, other neonicotinoids such as TMX have been shown to exert hepatotoxic and nephrotoxic effects in vivo. TMX (75WG) significantly impairs liver and kidney function, as evidenced by elevated biochemical markers of hepatic and renal injury and increased oxidative stress. Additionally, TMX induces histopathological damage in liver and kidney tissues, indicating a pronounced hepatotoxic and nephrotoxic effect [59]. Despite these consistent toxicological patterns, comparisons across studies are challenged by differences in compound structure, exposure durations, and assessment endpoints. Furthermore, few studies have systematically compared structurally distinct neonicotinoids under standardized conditions, limiting mechanistic generalization across the class.

### 3.4. Immunotoxic Effects of Neonicotinoids on Immune Cell Function and Viability

Immune cells exhibit differential sensitivity to neonicotinoid-induced toxicity. Neonicotinoid compounds have significant impacts on immune cell activity. Multiple studies have reported that neonicotinoids affect the function of immune cells, underscoring their potential immunotoxicity. For instance, Rymuszka et al. found that CLO (40 μM, 24 h) reduced the viability of human CD4+T lymphocytes by 37% while only causing a 14% reduction in neutrophil viability at the same concentration [24]. Walderdorff et al. investigated the immune response of human macrophages (THP-1) to IMI exposure, revealing that phagocytic activity was significantly suppressed only at a high concentration (100 mg/L) and after 24 h of exposure [44]. This high-dose dependence raises concerns about environmental relevance, and whether immunosuppression occurs under realistic exposure levels remains uncertain. In addition, Di Prisco et al. demonstrated that, in the presence of CLO, pro-inflammatory stimulation of human monocytic THP-1 cells with lipopolysaccharide (LPS) led to a marked reduction in TNF-α production and downregulation of a reporter gene under the control of the NF-κB promoter [65]. Another study similarly reported that CLO can impair antiviral immunity in honey bees by upregulating leucine-rich repeat proteins, thereby inhibiting the NF-κB signaling pathway and promoting the replication of Deformed Wing Virus (DWV) [66]. These findings suggest a conserved immunosuppressive mechanism via NF-κB inhibition. Cestonaro et al. found that IMI (600 mg/L flowable suspension for seed treatment, 600FS) exerts dose- and time-dependent immunotoxic effects on RAW 264.7 murine macrophages [67]. IMI significantly reduced mitochondrial membrane potential (Δψm), with a more pronounced loss at 500–1000 mg/L after 96 h than after 24 h. Moreover, high concentrations of IMI significantly increased the activity of mitochondrial complex II and its key enzyme succinate dehydrogenase, with more pronounced elevations observed after 24 h than 96 h. Costa et al. used the resazurin assay to assess the viability of Jurkat cells exposed to IMI (0.2, 2, and 20 μM) for 24 h and found no significant changes compared to untreated controls [68]. Furthermore, IMI has been shown to modulate immune responses in pigs through the cholinergic anti-inflammatory pathway and to suppress T cell proliferation and alter the structure of immune organs in mice [45,69]. In insect models, such as honey bees and red mason bees, thiacloprid has also been shown to significantly impair immune function [70,71]. Together, these findings illustrate cross-species immunotoxicity but are complicated by variations in exposure doses, immune endpoints, and model organisms, emphasizing the need for standardized comparative studies.

### 3.5. Epithelial Cell Damage and Barrier Function Disruption Induced by Neonicotinoids

Neonicotinoids disrupt epithelial barrier integrity across multiple organ systems. In intestinal Caco-2 cells, IMI at concentrations as low as 100 nM significantly decrease transepithelial electrical resistance (TEER), increase permeability, and are accompanied by reduced expression of tight junction proteins [72]. This barrier disruption exhibits a dose-dependent pattern, in which IMI (0.10–0.75 μg/mL) progressively reduces the levels of occludin and E-cadherin while simultaneously upregulating proinflammatory mediators such as TNF-α and inducible nitric oxide synthase (iNOS) [73]. Similarly, ACE reduces Caco-2 cell viability in a concentration-dependent manner, showing significant cytotoxicity at concentrations ranging from 75 to 350 μM, whereas lower concentrations (25–50 μM) maintain cell viability above 95% [74]. Respiratory epithelial cells display comparable sensitivity to neonicotinoid exposure. CLO exhibits strong cytotoxic effects on human bronchial epithelial cells (BEAS-2B), with half-maximal inhibitory concentration (IC_50_) values of approximately 0.67 mM across exposure durations of 24 to 120 h. Notably, after 120 h of exposure, CLO at only 0.0068 mM reduces cell viability to 80% [28]. In pulmonary A549 cells, IMI demonstrates an IC_50_ of 1.8 mM following 72 h of exposure [75]. The response of mammary epithelial cells to neonicotinoids varies depending on the cell type. In MCF-7 breast cancer cells, IMI significantly reduces cell viability within the concentration range of 1600 pM to 1.6 μM, concomitantly decreasing intracellular ATP levels (160 pM–1.6 μM) and increasing ROS production at 1.6 nM. In contrast, non-tumorigenic MCF-12A cells exhibit inhibited proliferation only at higher concentrations (1.6 μM), with ROS levels decreasing within 16–160 μM, indicating a cell type-specific response in mammary tissue [76]. The contrasting responses observed between tumorigenic and non-tumorigenic cells highlight the critical role of cellular status in the identification of toxicological mechanisms. Cancer cells may exhibit greater sensitivity to pesticide exposure due to mitochondrial dysfunction and the activation of stress response pathways; however, such responses may not accurately reflect those of healthy tissues. Therefore, future studies should systematically compare cell models with different differentiation states and tissue origins to investigate variations in metabolic capacity, transporter expression, and antioxidant defense systems. This approach is essential to avoid overestimation or underestimation of the toxic effects of neonicotinoids. Prostate epithelial cells (WPM-Y.1) show high sensitivity to IMI, with an IC_50_ of 0.023 mM after 24 h of exposure. Mechanistically, IMI exposure increases lactate dehydrogenase (LDH) activity and MDA levels while depleting glutathione (GSH) content and glutathione S-transferase (GST) activity, suggesting cytotoxicity mediated by oxidative stress. Ultrastructural analysis reveals significant cellular damage following pesticide treatment [77]. However, most current studies rely on single time-point biochemical measurements, which may underestimate transient or reversible effects induced by pesticide exposure.

### 3.6. Toxicological Effects of Neonicotinoids on Other Cell Types and Organ Systems

Several studies have demonstrated that neonicotinoids can influence lipid metabolism. Park et al. reported that treatment with 10 μM and 20 μM IMI increased lipid accumulation in 3T3-L1 adipocytes and significantly upregulated the expression of key regulators involved in adipocyte differentiation and lipogenesis [78]. These findings suggest that IMI may disrupt normal adipogenesis, leading to enhanced fat accumulation. Similarly, Mesnage et al. assessed the adipogenic potential of seven major neonicotinoids (TMX, IMI, CLO, FLU, DIN, NIT, THI) using 3T3-L1 cells [79]. Among these, only IMI induced triglyceride accumulation at concentrations starting from 50 mg/L, indicating its unique adipogenic effect. Additionally, FIP has been shown to promote lipid accumulation; Sun et al. found that 10 μM FIP treatment increased fat accumulation in 3T3-L1 adipocytes and stimulated the expression of critical adipogenic regulators such as CCAAT/enhancer-binding protein alpha (C/EBPα) and peroxisome proliferator-activated receptor gamma (PPARγ), as well as lipogenic enzymes, including acetyl-CoA carboxylase and fatty acid synthase [80]. Neonicotinoids also exert effects on fibroblast cells. Sevim et al. investigated the dose- and time-dependent impact of IMI on acetylcholinesterase (AChE), LDH, and GSH levels in the L-929 fibroblast cell line [81]. Their results revealed a significant dose-dependent increase in LDH levels in cells treated with 250 and 500 ng IMI compared to controls. GSH levels showed a nonsignificant dose-dependent decrease, with the lowest GSH observed in the 500 ng IMI group relative to controls. No significant changes were found in AChE activity among the groups. These findings suggest that high doses of IMI may induce oxidative stress in fibroblast cells. To further assess whether IMI induces cytotoxicity and morphological alterations in normal human cells, Eriksson et al. evaluated IMI (70WG) effects on human AG01518 fibroblasts [52]. They found that IMI caused cytotoxicity at concentrations above 0.8 mM, as determined by the MTT assay, and observed vesicular accumulation of intracellular material at concentrations exceeding 0.4 mM. Kara et al. examined the cytotoxicity of ACE on the AR42J pancreatic cell line, reporting a dose-dependent decrease in cell viability, with an IC_50_ value of 12.61 mM based on the MTT assay [82]. Gomez et al. analyzed the viability of HTR-8/SVneo cells following exposure to ACE at concentrations of 0.1, 1, 10, and 100 μM, demonstrating differential modulation of viability with a significant reduction observed at 100 μM [83]. Baysal et al. investigated the toxicity of IMI and ACE on HT-29 cells [29]. After 72 h of individual compound exposure, IMI significantly reduced cell viability at 3200 and 4000 μM, while ACE decreased viability at 2400, 3200, and 4000 μM compared to controls. Notably, combined exposure to IMI and ACE in a 1:1 mixture at concentrations of 200, 300, 400, and 800 μM also markedly decreased cell viability relative to controls. Furthermore, all treatment groups exhibited a reduction in mitochondrial membrane potential, indicating mitochondrial dysfunction.

## 4. Public Health Implications

A recent biomonitoring study by Thompson et al. detected neonicotinoid metabolites in 92% of urine samples from a general population cohort, with concentrations ranging from 0.1 to 8.4 ng/mL, indicating widespread human exposure [84]. Increasing evidence suggests that neonicotinoid insecticides exhibit cytotoxicity across various cell types and organ systems, posing significant implications for public health. Epidemiological studies have begun to uncover associations between neonicotinoid exposure and adverse health outcomes. For example, a mother–child cohort study from Taiwan reported that childhood exposure to neonicotinoids, particularly in boys aged 4–6 years, was associated with reduced scores in the Fluid Reasoning Index of cognitive assessments, suggesting potential detrimental effects on neurodevelopment [85]. Additionally, a study conducted in southern China detected widespread presence of neonicotinoid parent compounds and metabolites in maternal serum, with exposure levels associated with decreased maternal free thyroxine (FT4) and changes in neonatal birth outcomes, indicating potential endocrine-disrupting effects and risks to fetal growth [86]. Another nested case-control study found that elevated mid-pregnancy serum concentrations of neonicotinoids—particularly nitro-substituted compounds like imidacloprid—were associated with an increased risk of fetal septal defects, although statistical significance has yet to be firmly established [87]. The cellular mechanisms reviewed in this article provide biological plausibility for these epidemiological findings. Disruptions in neuronal calcium homeostasis and mitochondrial function may underlie the observed neurodevelopmental effects, while oxidative stress and DNA damage may explain potential carcinogenic or teratogenic outcomes. Given the widespread exposure through food, water, and environmental pathways, even low-dose chronic exposure deserves close attention, particularly in light of the heightened vulnerability of developing organisms and the potential for mixture effects with other environmental contaminants.

## 5. Conclusions

This review has integrated emerging evidence on the cellular mechanisms underlying neonicotinoid-induced cytotoxicity, revealing that their biological effects extend beyond classical neurotoxicity. Current findings suggest that disruptions in oxidative stress responses, DNA stability, calcium signaling, and endoplasmic reticulum function—along with evidence of cell-type specific vulnerabilities—may contribute to the observed toxicological profiles. As agricultural systems strive to meet rising global food demands, a key challenge will be to mitigate potential risks to human and environmental health without compromising productivity. To address this challenge, sustained interdisciplinary collaboration will be essential for developing risk assessment frameworks that are both scientifically robust and aligned with practical agricultural needs. A deeper understanding of neonicotinoid mechanisms may ultimately help refine exposure assessments, identify sensitive populations, and support the development of safer, more sustainable pest management alternatives.

## Figures and Tables

**Table 1:** Overview of major neonicotinoid insecticides, typical formulations.

Active Ingredient	Example Formulation
Imidacloprid	200SL (soluble concentrate), 70WG (water-dispersible granules), 25WP (Wettable Powder)
Thiamethoxam	25WG, 350FS (flowable concentrate for seed treatment)
Acetamiprid	20SP (soluble powder), 5SL
Clothianidin	600FS
Thiacloprid	480SC (suspension concentrate)
Dinotefuran	20SG (soluble granules), 10WP
Nitenpyram	Oral tablets (mainly for veterinary use)
Flupyradifurone	200SL
Cycloxaprid	Common in mixture formulations
Sulfoxaflor	240SC, 50WG

## Data Availability

Not applicable.
